# Rigid ureteroscopic lithotripsy in the lateral decubitus position for upper urinary tract stones

**DOI:** 10.1186/s12894-022-00977-x

**Published:** 2022-02-23

**Authors:** Jinqing Zhang, Binbin Li, Gang Li, Zengshi Yang, Ning Ye, Yihao Liu, Hongbing Zhuo, Jingfan Hong

**Affiliations:** 1 Department of Urology, Affiliated Xiaolan Hospital, Southern Medical University, Zhongshan, Guangdong China; 2 Department of Urology, Affiliated Foshan Hospital of SUN Yat-Sen University, The First People’s Hospital of Foshan, Foshan, Guangdong China; 3 Department of Urology, Hospital of Traditional Chinese Medicine of Zhongshan, Zhongshan, Guangdong China; 4 Department of Urology, Dongguan Kanghua Hospital, Dongguan, Guangdong China

**Keywords:** Ureteral calculi, Body position, Ureteroscopy, Lithotripsy, Stone-free rate

## Abstract

**Background:**

The current study aimed to assess a novel ureteroscopic technique developed for treating upper urinary calculi based on a specially designed lateral decubitus body position that could avoid stone loss by adjusting to the effects of gravity.

**Methods:**

This retrospective study examined patients with upper urinary calculi who were surgically treated from November 2008 to January 2020, using a new body position and a rigid ureteroscope. Clinical outcomes, stone-free rates, operative times and complications were evaluated, and factors that could influence treatment success were determined.

**Results:**

In total, 1080 patients were included, and 1145 operations were performed. The maximum calculus diameters were 11.22 ± 5.01 mm. Operative times were 48.60 ± 27.44 min. A total of 1042 cases were successfully treated, with a stone-free rate of 91.00%. Multivariate analysis showed that female sex (OR = 2.135, 95% CI 1.332–3.422, *P* = 0.002), thin scope standby (OR = 1.643, 95% CI 1.074–2.514, *P* = 0.022), laser lithotripsy (OR = 5.087, 95% CI 2.400–10.785, *P* = 0.000) and stone size (OR = 0.946, 95% CI 0.912–0.981, *P* = 0.003) were independently associated with stone-free outcomes. In total, 2 ureteral perforations, 2 ureteric avulsions and 4 urosepsis cases were observed, but were all cured without sequelae.

**Conclusions:**

Ureteroscopic lithotripsy in the lateral decubitus position is a safe and effective technique for treating upper urinary tract calculi, especially upper ureteral calculi.

## Background

Urinary stones constitute the most common pathology affecting the urinary tract, and elevate the risk of chronic kidney diseases, end-stage renal failure, cardiovascular disease, diabetes and hypertension [1]. The treatment of urinary tract stones has been greatly improved, and new technologies and instruments provide more choices for managing all types of calculi. For upper urinary tract stones, extracorporeal shockwave lithotripsy (ESWL), rigid ureteroscopic lithotripsy, laparoscopic lithotomy, percutaneous nephrolithotomy (PCNL), flexible ureteroscopic lithotripsy, and open surgery, which is not usually necessary, are available [2].

**Fig. 1:**
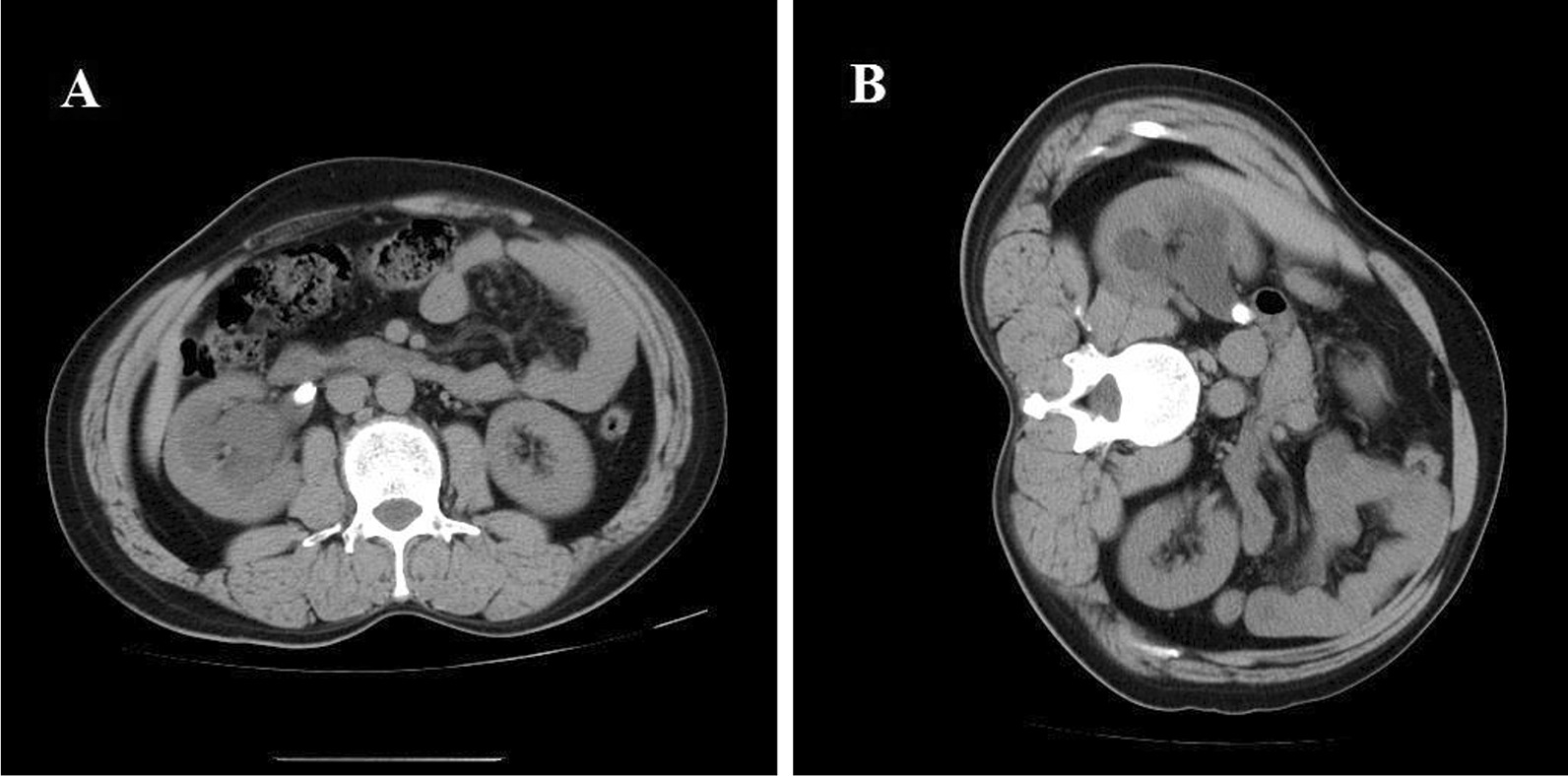
Renal pelvis shown on computed tomography (CT) images for different body positions. **A** In the supine position, the renal pelvis forms an angle that tends to guide the stone into the kidney if loosened during the operation. **B** In the lateral position, the left ureteropelvic junction moves to the lowest point. The stone tends to not migrate into the kidney and would fall back if it does

Ureteroscopy lithotripsy is noninvasive, relatively effective, safe and inexpensive; however, this approach often fails because of the stone’s retrograde migration, especially when performed with a rigid scope and a ballistic lithotripter [3, 4]. In the supine or lithotomy position, the normal rotational axes of the kidney make an angle of approximately 30° with the horizontal plane [5]. The pelvis and calices are lower than the ureter; therefore, retrogradely migrating ureteral stones will tend to fall into the kidney along the slope of the pelvis (Fig. 1A). Thus, the stones will no longer be reachable by the instrument. Using a laser, this problem becomes slightly less prominent, but ureteral stone retropulsion still occurs [6].

In recent years, flexible ureteroscopy has become a research focus and has been developed rapidly [7, 8]. Indeed, the flexible ureteroscope has many advantages, including treating a ureteral stone even after progression into the calix [7, 9]. However, this method has some unignorable limitations. For example, the durability of the flexible ureteroscope is not satisfactory, and it requires frequent repairs [10, 11]. Additionally, the cost of the scope and accessories, as well as repairs, is high [12]. To avoid maintenance and repair costs, single-use scopes and ball-tip laser fibers have been developed [8, 13]. Despite the above efforts, the cost of flexible ureteroscopy remains higher than that of rigid ureteroscopy. Furthermore, a flexible ureteroscope is not as easy to control as a rigid ureteroscope, especially for inexperienced operators [14]. Most flexible scopes have larger diameters than rigid scopes, and usually require sheaths, rendering them more difficult to pass through the ureter [15]. Related complications, such as tears and rupture, are more common with flexible scopes than rigid scopes [15]. Finally, a flexible scope is more difficult to clean than a rigid scope [16].

PCNL can also be used to treat upper ureteral stones, and has undergone innovative improvements [17]. However, PCNL obviously incurs high risk, results in multiple complications and has a high cost [18]. Using baskets or balloons is another way to avoid upper ureteral stone migration. However, this approach poses specific problems. For example, the basket or balloon cannot easily be placed in the proper position and readily causes injury of the ureteral wall, and may even be destroyed by the lithotripsy instrument [19]. Radioactive exposure is also an issue associated with these techniques.

Therefore, the present study aimed to evaluate a new ureteroscopic technique developed to treat upper urinary calculi.

## Methods

### Study design and participants

This was a retrospective study assessing more than 1000 cases of upper urinary calculi treated using the newly developed method, from November 1, 2008 to January 31, 2020, in Xiaolan People’s Hospital of Zhongshan, Southern Medical University. Upper urinary stones in this study referred to one or multiple upper ureter or pelvic stones diagnosed by computed tomography (CT) according to current standards [20]. The inclusion criteria were age > 16 years, ureteroscopic lithotripsy performed in the lateral decubitus body position and 1-month postoperative follow-up. The exclusion criterion was incomplete clinical and/or follow-up data. The study was approved by the Medical Ethics Committee of Xiaolan People’s Hospital of Zhongshan. The requirement for informed consent was waived because of the retrospective nature of this study.

### Data collection

Here, we applied a new treatment procedure, i.e., rigid ureteroscopic lithotripsy with the patient in a specially designed lateral decubitus body position.

**Fig. 2:**
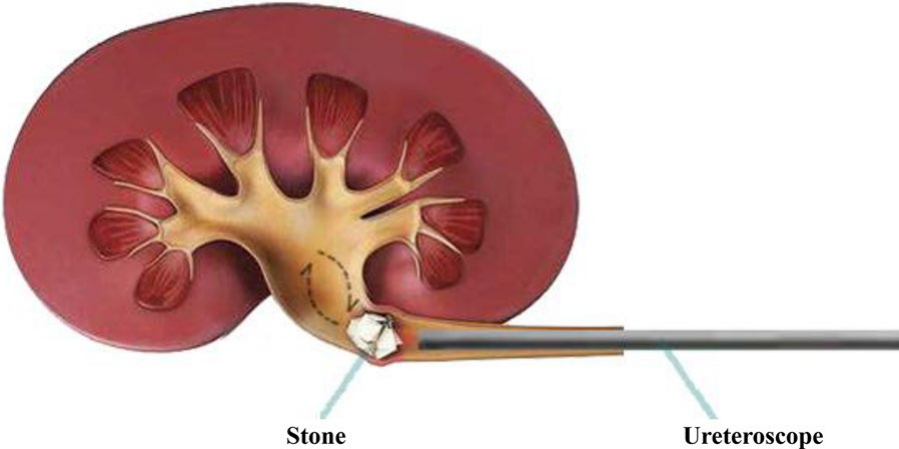
Schematic diagram of the mechanism of ureteroscopic lithotripsy in the lateral decubitus position

The key feature of the technique is patient placement in the lateral decubitus position on the ipsilesional side. Thus, the junction between the pelvis and ureter moves to the lowest level of the upper urinary tract (Fig. 1B); this location is usually reachable by rigid ureteroscopes. A stone migrating proximally during the operation would stay there instead of falling into the kidney. Even if a stone bounces into the kidney temporarily by the impact of lithotripsy, it would fall back under the influence of gravity most of the time (Fig. 2). Therefore, the escape of a stone from the scope is theoretically impossible.

**Fig. 3:**
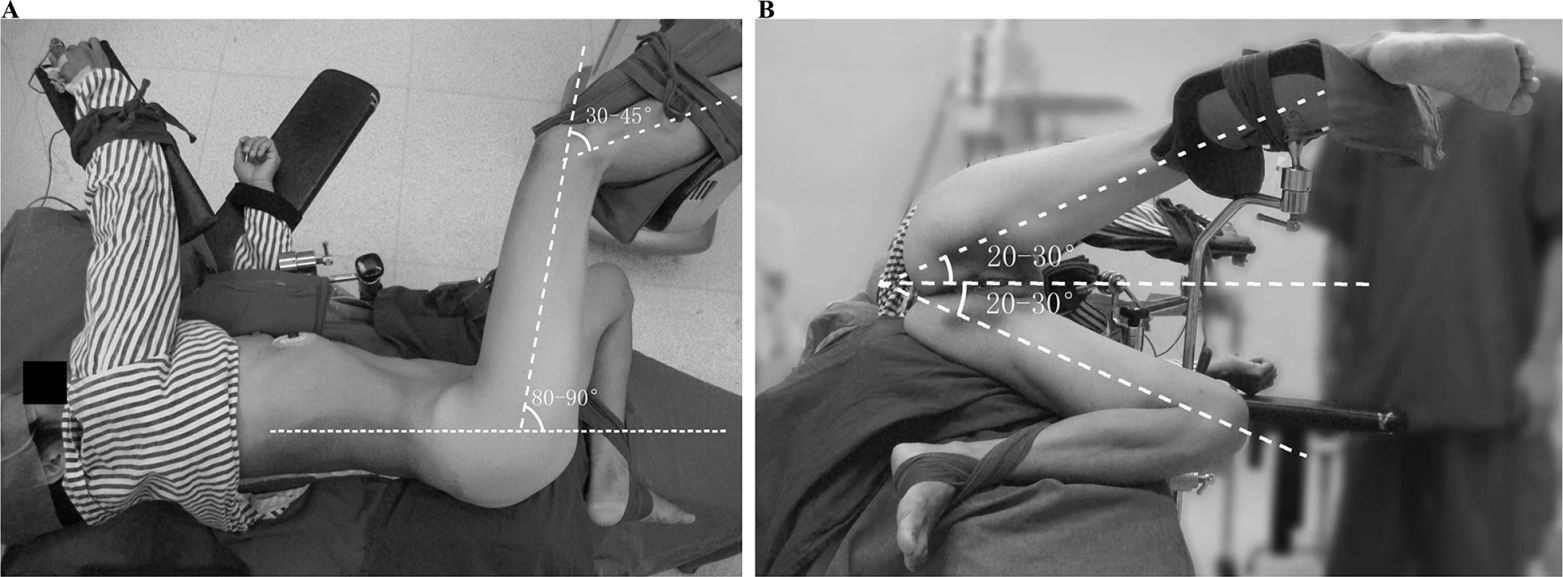
The unique lateral position that was designed for the ureteroscopy

The body position was designed to be equivalent to the lithotomy position rotated by 90°. After anesthesia, mostly general and sometimes combined spinal-epidural anesthesia, the patient was placed in the lateral decubitus position lying on the ipsilesional side with the coxa at the edge of the operating table. For the ipsilesional leg, the coxa joint was flexed to 80°–90° and abducted to 20°–30°, with the knee joint flexed to 30°–45°. For the contralesional leg, the coxa joint was flexed to 60°–80° and abducted to 20°–30°, with the knee joint flexed to the utmost extent. The torso was kept straight, and the spine was slightly extended. The upper extremities were fixed as in the common lateral position. The axillary fossa and the area around the greater trochanter were carefully cushioned. The patient was fixed, well cushioned and placed in appropriate comfort using the operating table accessories (Fig. 3).

The most commonly used ureteroscopes were type 8703.534, 8/9.8 Fr (Richard Wolf, Germany). Stones could be chased to the kidney, sometimes even to the upper calix. Ballistic lithotripsy and a laser were both used. The laser was obtained from the SRM-H3B holmium:yttrium aluminum garnet (Ho:YAG) laser system (Raykeen, China), and typically used at a frequency of 20 Hz and an energy of 1.6 W. A postoperative stent of 5 Fr was routinely employed for each case.

To avoid severe surgery-related infections, the patients were carefully examined and prepared preoperatively. They were required to exhibit no fever for more than 3 consecutive days. In addition, routine urinalysis was expected to show fewer than 2+ leukocytes and no nitrite. Urine culture or recheck was expected to be negative. Otherwise, the patients were treated with antibiotics until they met all the above requirements. Antibiotics were routinely administered from 0.5 h before every operation until 48 h postoperatively. Furthermore, if the patient developed a fever, antibiotic treatment was not stopped until 3 days after the fever subsided.

Since a narrow ureter was found to impede the operation, thin ureteroscopes started to be applied in November 2013. Thin scopes could be moved under conditions in which normal-size scopes were blocked by the part with too small a calibre. The first scope was 6.0 Fr (Shoelly®), and type 8702.534, 6.0/7.5 Fr scopes (Richard Wolf) were later used.

### Postoperative and follow-up evaluations

One month post-operatively, before removal of the stent tube, abdominal radiography or CT was routinely performed to assess remaining calculi. The “stone-free” status was defined as no remainder ≥ 2 mm on the operated side; otherwise, the treatment was considered to have failed even if the stone was partially treated.

### Statistical analysis

Continuous variables were expressed as the mean ± standard deviation, and were compared by Student’s *t* test. Categorical variables were expressed as numbers and frequencies, and analyzed by the chi-square test. Univariate and multivariate logistic regression analyses were performed to determine independent risk factors for stone-free outcomes. Statistical Package for Social Sciences 17.0 (SPSS 17.0, SPSS Inc., Chicago, IL, USA) was used for data analysis.

## Results

### Characteristics of the participants

In total, 1145 operations in 1080 patients with a history of urinary calculi ranging from 1 day to 13 years were included in this study. All baseline features are summarized in Table 1.

**Table 1:** Clinical and demographic characteristics of the study population

	Total operations (n = 1145)	Success (n = 1042)	Failure (n = 103)	*p*
Age (years)	45.32 ± 13.48 (17–87)	45.18 ± 13.39 (17–87)	46.74 ± 14.34 (22–82)	0.264
Gender				0.030
Male	698	621	77	
Female	447	421	26	
Stone size (mm)	11.33 ± 5.12 (3–38)	11.22 ± 5.01 (3–37)	12.49 ± 6.02 (4–38)	0.017
Number of stones	1.27 ± 0.79	1.27 ± 0.79	1.24 ± 0.73	0.410
Side				0.805
Left	587	533	54	
Right	558	509	49	
Location*				0.851
Renal pelvis	174	159	15	
Upper ureter	971	883	88	
Lithotripsy				0.00
Ballistic	806	711	95	
Laser	339	331	8	
Operative time (min)	–	48.60 ± 27.44 (10–210)	114.84 ± 82.66 (20–465)	–
Complications**				
Ureter perforation	2	2	0	–
Ureteral avulsion	2	0	2	–
Leg numbness	1	1	0	–
Urosepsis	4	4	0	–

Data are presented as the mean ± SD, with range or n

^*^Location defined as that of the highest stone treated in the operation

^**^Data of fever were shown in Table

### Treatment outcomes

The total number of operations (1145) differed from that of patients (1080) because 59 and 3 patients underwent the operation twice and thrice, respectively, at intervals ranging from 1 week to more than 3 years. While summarizing data, such as age, operative time and patient gender, operations performed in the same individuals were treated as different cases. The maximum stone diameter means the longest diameter for single stone or the sum of the longest diameters for multiple stones. They ranged from 3 to 38 mm, averaging 11.33 ± 5.12 mm. There were 698 operations performed in males and 447 in females, and 587 and 558 procedures were performed on the left and right sides, respectively. Single ureteral (883 cases), single renal (65 cases), multiple renal (72 cases) and single renal/ureteral (22 cases) stones were successfully treated. The mean operative time (from scope introduction to catheterization after lithotripsy) was 48.60 ± 27.44 min. No difficulties were observed to be caused by the position with respect to the anesthetist for general or intraspinal anesthesia.

Meanwhile, a total of 103 cases could not be treated successfully by the current procedure and subsequently underwent PCNL, ESWL or open surgery, depending on the specific disease condition and patient preference. The stone-free ratio was 91.00% (1042/1145).

The reasons for failure (Table 2) were analyzed based on the surgical occurrence and postoperative iconographic review. The most frequently recorded reason was ureter narrowing. The second most common was loss of the stone or its fragment in the kidney, although the novel technique attempted to avoid this. Three failed cases were not included in the analysis because the related reasons (instrument malfunction, unidentifiable ureteral orifice that remained unidentifiable in the lithotomy position due to severe cystitis, and ureter deformity that was not related to position) were not considered to be associated with the technique at all.

**Table 2:** The reasons for failed cases

Reason	Cases
Narrowing of the ureter	54
Stone or a part thereof trapped in the kidney	22
Ureteral tortuosity	10
Oversized/hard stone*	8
Pelvis deformity induced by severe hydronephrosis**	4
Blockage by polyps secondary to lithiasis	2
Ureter avulsion	2
Pyonephrosis***	1

^*^Rendered the operation impossible to complete in a reasonable time

^**^The stone sank to the lowest point of the enlarged pelvis and was unreachable by the ureteroscope

^***^The operation was aborted due to concerns about possible sepsis

### Factors associated with treatment outcomes

There were 77 failed cases among the 698 males and 26 failed among the 447 females. In total, 54 and 49 failed cases were recorded among the 587 left-sided and 558 right-sided procedures, respectively. Multivariate analysis (Table 3) showed that female sex (OR = 2.135, 95% CI 1.332–3.422, *P* = 0.002) was significantly associated with stone-free outcomes.

**Table 3:** Logistic regression analysis of risk factors for a stone-free outcome (success)

Variables	Univariate analysis			Multivariate analysis		
	OR	95% CI	*P*	OR	95% CI	*P*
Gender (male as ref)	2.008	1.266–3.185	0.003	2.135	1.332–3.422	0.002
Side (left as ref)	1.052	0.702–1.578	0.805	1.006	0.662–1.527	0.979
Thin scope standby (no as ref)	2.029	1.374–3.114	0.000	1.643	1.074–2.514	0.022
Laser lithotripsy (ballistic as ref)	5.528	2.656–11.509	0.000	5.087	2.400–10.785	0.000
Stone location (renal pelvis as ref)	0.947	0.534–1.679	0.851	0.859	0.472–1.565	0.620
Stone size (mm)	0.958	0.925–0.993	0.018	0.946	0.912–0.981	0.003
Number of stones	1.054	0.802–1.386	0.706	1.035	0.788–1.359	0.806

Thin ureteroscopes were used 68 times, including in 15 failed cases. This procedural modification greatly improved the success rate from 86.9% to 93.5%, a statistically significant difference as assessed by the chi-square test (P < 0.001). Multivariate analysis (Table 3) supported this significance (thin scope standby: OR = 1.643, 95% CI 1.074–2.514, *P* = 0.022) for stone-free outcomes.

Because of stone hardness or economic reasons, some patients underwent laser treatment. Multivariate regression analysis (Table 3) showed that laser lithotripsy was independently associated with stone-free outcomes (OR = 5.087, 95% CI 2.400–10.785, *P* = 0.000).

### Complications

For technical reasons, postoperative temperatures could be reviewed only from December 2013 to January 2020 in 641 cases. A total of 102 patients (15.9%) developed a fever (Table 4). There were a total of 4 urosepsis cases in this study, including 1 occurring before December 2013. The patients were all subsequently cured by conservative treatment.

**Table 4:** Postoperative fever (Dec. 2013–Jan. 2020)

Temperature*	Normal	37–38.4 °C	38.5–39.9 °C	≥ 40 °C
Cases	539	68	26	8**
Rate	84.9%	10.6%	4.1%	1.2%

^*^Highest axillary temperature reached

^**^Including 3 confirmed cases of urosepsis

Information of other complications could be seen in Table 1. Two cases of notable ureter perforation occurred and were treated with ureteric stenting assisted by urethral catheterisation. Urethral catheterization were removed 14 days after operation and ureteric stent tube, 1 month. Recoveries were proved by imagining examinations. Two cases of ureteral avulsion, which occurred at the joint between the ureter and the bladder, were cured by open surgery.

No remarkable bleeding was observed perioperatively. One patient required blood transfusion because of sepsis instead of surgical bleeding. Leg numbness on the same side of the stone appeared in one case, which was believed to be a result of nerve compression, and was relieved 1.5 months later by conservative therapy. No visceral, vascular, urethral or bladder injuries were found, and no mortality was observed.

## Discussion

With the current technique, a sizeable proportion of upper urinary stone cases, especially those involving upper ureteral stones, can be treated by using a rigid scope. Thus, the consumption of flexible scopes can be reduced, resulting in lower healthcare costs. This is especially significant for most hospitals in developing countries that cannot afford flexible scopes.

Since lateral decubitus surgery has been a routine operation in our hospital for a long time, there were no comparative data on ureteroscopic lithotripsy in the lithotomy position for upper urinary stones in the same center. A study on semirigid ureteroscopy with pneumatic lithotripsy for 75 cases of large proximal ureteral calculi showed an initial stone-free rate of 90.6%,but in 14 patients (18.6%) complete stone clearance was not achieved through primary operation because of migration of the entire or partial stone to the kidney [21]. In a study for semirigid ureteroscopic lithotripsy with laser treating upper ureteral stones, the stone-free rate was reported to be 86.6% [22]. Despite that they both used dilation instruments, the stone-free rates are both lower than the level in this study.

The current study covered such a long time that there were some opportunities to observe beginners learning to perform ureteroscopic lithotripsy in the lateral decubitus position. Despite the initial difficulties in passing the ureteroscope through the male urethra and finding the correct orifice, a skilled ureteroscope user could become accustomed to the technique after 2 or 3 times of applications under the guidance of a veteran.

According to our observations, the lateral position was fairly endurable for patients because it is as comfortable as the supine position and may even be better for some patients, such as individuals with obesity or spinal disease.

It is not surprising that the most common reason for failure of this technique was ureter narrowing. The narrowest part of the ureter is usually the terminal portion [5]. However, the base part of a ureteroscope is thicker than its tip. Because the operation was designed to treat stones at the upper ureter or renal pelvis, the scope had to travel very deep. Consequently, the thickest part of the scope had to proceed through the narrowest part of the ureter, which certainly has the tendency to get stuck. As expected, the use of thin ureteroscopes has greatly improved the success rate of the operation.

Migration of a stone in ureteroscopic lithotripsy procedures, was reported to occur in 2–60% of the cases [23]. It is a challenges to deal with during ureteric stone management, especially proximal ureteric stones and influenced by several factors such as the pressure of irrigation fluid, degree of proximal ureteral dilation, stone site, the degree of stone impaction, lithotripter type, anti-retropulsive devices and experience of the surgeon [24]. In the current study, migration of a stone was not deliberately prevented so that it was commonly seen. However, due to the advantages of this surgical technique, it had little influence on the success of the operation. Many upper ureteral calculi were finally fragmented in the renal pelvis.

Although the body position applied in the present study was designed to prevent migrating stones from dropping into the kidney, cases that some stones did not return after falling into the kidney in the lateral position still occurred. By comparing postoperative and preoperative conditions, the main reason was considered to be the deformity of the collecting system. After entering the kidney, the stone was captured in a concavity formed by an enlarged calix or the pelvis.

When the current surgical approach was originally designed, it was assumed that the lithotripsy option would have no effect on the success rate of the operation. Surprisingly, statistical results showed that the success rate of laser lithotripsy was actually higher than that of ballistic lithotripsy. The proposed reason was that the laser could powder the stones, while ballistic lithotripsy makes larger particles that easily accumulate, interfering with the observation and resulting in the omission of large stone fragments. The larger the stone is, the more difficult it is to shatter it thoroughly and evenly. This may also explain why stone size was negatively correlated with the stone-free rate in this study.

The prostatic urethra is relatively fixed, harder than the female urethra, and not aligned with the ureter, indicating differences between the male and female urinary tracts [25]. Therefore, while performing operations in male patients, the ureteroscope is not as movable as it is in females, which makes it more difficult to address troublesome conditions such as ureteral tortuosity, pelvic deformity and stone disappearance. This may explain the higher success rate in female patients than in males.

The current technique has a notable shortcoming. As mentioned above, it requires very deep insertion of the scope. Consequently, outflow was not easy during operation, which made the pelvis pressure high. Therefore, infection should be carefully monitored and prevented. In this study, infection was carefully prevented. Altogether, only 4 urosepsis cases were recorded. In addition, the treatment outcome was acceptable, with no mortality.

Ureteroscopic lithotripsy in the lateral decubitus position has been applied as a routine approach to treat upper ureteral or renal stones since 2003 in the Affiliated Xiaolan Hospital of Southern Medical University. In 2006, this technique was reported in its early stage [7]. Unfortunately, the present study was a single-center retrospective trial with no control group, and many factors could not be randomized. However, its large sample size could partly compensate for this issue. Further prospective studies are warranted to confirm these findings. However, with the continuous progress of flexible ureteroscopy and other urological technologies, an increasing number of better options will be available in the future.

## Conclusions

Ureteroscopic lithotripsy in the lateral decubitus position is a safe, effective and relatively economical technique for treating upper urinary calculi, especially upper ureteral calculi. Moreover, it yields a satisfactory stone-free rate. This procedure is strongly recommended for upper urinary calculi but is not suitable for complicated or large renal stones. Overall, ureteroscopic lithotripsy in the lateral decubitus position is more suitable for female patients and preferentially involves the use of a laser.

## Data Availability

The data set supporting the results of this article is included within the article. The datasets used and/or analyzed during the current study are available from the corresponding author on reasonable request.

## Abbreviations

ESWL: Extracorporeal shockwave lithotripsy
PCNL: Percutaneous nephrolithotomy
